# Retraction: Economic affordability of food as a component of the economic security of Ukraine

**DOI:** 10.1371/journal.pone.0314383

**Published:** 2024-11-20

**Authors:** The PLOS ONE Editors

After this article [1] was published, the following concerns were raised:

- The article [1] contains citations, including by the last author, that do not support the statements in which they are cited.
- There are concerns about competing interests and peer review.
- A member of the *PLOS ONE* Editorial Board reviewed the article [1] and noted concerns about the data and analyses that were not fully resolved in post-publication discussions with the corresponding author, including a lack of unit root and cointegration tests, concerns about the interpretation of R^2^ and unclear reasoning for the estimation strategy.

The *PLOS ONE* Editors retract this article [1] due to the above concerns. We regret that the issues were not addressed prior to the article’s publication.

LV, TLM, and EY did not agree with the retraction. TGM and ZL either did not respond directly or could not be reached.
